# Supplementary material for: Mobile phones: The effect of its presence on learning and memory
Source: PLoS One. 2020 Aug 13;15(8):e0219233. doi: 10.1371/journal.pone.0219233 (PMC7425970; doi:10.1371/journal.pone.0219233)
Supplement: Attachment — Submitted filename: Response to Reviewers.docx [file pone.0219233.s001.docx]

18 October 2019

Dear Academic Editor,

We would like to express our thanks and gratitude for the helpful comments raised in our paper. Below is a point-by-point response to each comment/question. Please note that the line numbers and pages is taken from the clean version of the revised manuscript. The citations and references are also taken from the clean manuscript, and as such the numbering of the references will be off in this letter.

Best regards,

C Tanil & MH Yong

----

Reviewers' comments:

*Please use the space provided to explain your answers to the questions above. You may also include additional comments for the author, including concerns about dual publication, research ethics, or publication ethics. (Please upload your review as an attachment if it exceeds 20,000 characters)*

Reviewer #1: The present study examined the mnemonic consequences associated with the presence of a smartphone. Overall, the authors found that participants without their cellphones had higher accuracy scores than those who had their cell phones present. They also found a negative correlation between accuracy and "phone conscious thought."

Overall, I think this is an interesting area of research. However, the following issues need to be addressed before I can recommend publication. I will start with the larger issues before moving to the smaller issues:
** we thank you for your insightful comments. We have addressed each point in subsequent pages.

Larger issues

-Probably the biggest issue I found was the interpretation of their results. For example, on pg. 17, the authors state that "Although we did not find a significant relationship between SAS to memory accuracy, our measurements to 'phone conscious thought' is more relevant and meaningful because it measured participants separation anxiety..." This simply cannot be true: First, the question representing" phone conscious thought" asks "During the memory test how often do you think of your smartphone?" What does this even mean, exactly? How did participants interpret this question? Either way, I think it is quite a stretch to consider this anxiety. And, second, the SAS included a "'Withdrawal' sub-factor [that] describ[ed] the feeling of anxiety when separated from one's mobile phone." (pg. 9), but the authors found no significant correlations for any of the subfactors. Thus, not sure how a vague question about thoughts better represents anxiety than the specify subfactor of the SAS.

*** We thank the reviewer for this comment.

We should explain our reasoning for asking ‘phone conscious thought’ question. In Ward et al.’s study, they included three questions post-task, and we used two out of three questions. The two questions were (1) phone conscious thought “how often were you thinking about your cellphone” and (2) “…to what extent they believed their phones affected their performance and attention spans” (p. 145). The third question was about phone location, and we did not ask this question because we only had two locations and is a pointless question in our study. Ward et al. found that as smartphone salience increases (measured by the 3 questions), available cognitive capacity decreases – which is an indication that this particular question is meaningful to tap endogenous interruptions due to smartphone-related usage throughout the task. Even though the participants in both LS and HS conditions were not allowed to use their phone, their high phone use (average use per day in our sample was 8.16 hours, and 47% participants or 56 out of 119, are considered as addicted when compared to Kwon’s sample) might have evoked such thoughts, as suggested by Wilmer and Chien (2017) in their review. Some participants consider their smartphone as a ‘limb’ and losing this ‘limb’ is more common and has powerful effects than previously thought.

In Kwon et al.’s paper, the authors described the withdrawal sub-factor as “…involves being impatient, fretful, and intolerable without a smartphone, constantly having one’s smartphone in one’s mind even while not using it, never giving up using one’s smartphone, and becoming irritated when bothered while using one’s smartphone…” (p. 7). The 6 specific questions are as follows:

1. Won’t be able to stand not having a smartphone
2. Feeling impatient and fretful when I am not holding my smartphone
3. Having my smartphone in my mind even when I’m not using it
4. I will never give up using my smartphone even when my daily life is already greatly affected by it.
5. Getting irritated when bothered while using my smartphone
6. Bringing my smartphone to the toilet even when I am in a hurry to get there

One of the bigger challenges in using self-reported survey such as SAS is that these questions brings further attention to their behaviour which may then indirectly affects their response behaviour “social desirability” and/or inability to recall the frequency of such behaviour. Having the phone conscious thought is more spot-on and without the risk of both social desirability (negative terms such as impatient, fretful, irritation) and asking individuals to reflect on their past behaviour.

As to what our participants thought of seeing this question, we think that this is a simple straightforward question. We have since added more information about phone conscious thought in Abstract (page 2, line 6), Introduction (page 6, line 91-97), and Discussion (page 19, line 347-353).

-Additionally, the authors suggestion that the decrease in positive affect is the result of "prohibited usage/or separation from their phone" (pg. 18). But the authors have no data to support this. For all they know, the participants had a decrease in positive affect simply because they were participating in a study since both groups exhibited this.

*** We thank the reviewer for this comment. Indeed, both LS and HS groups experienced a decrease in PA. We realised that the sentences were misleading, and we apologise for the confusion. We have since reworded the sentences, see below and also on page 20, Line 364-369.

“While both groups showed a decrease in PA after completing the tasks, it is possible that the reduced PA is likely to have stemmed from the prohibited usage “HS” and/or separation from their phone “LS”. This is consistent with Cheever et al. (15), whose participants reported increased anxiety over time when separated from their phones and with Clayton, Leshner and Almond (18) findings, where participants were unable to use their phone.”

-In terms of the procedure, I'm a little concerned that only the "HS group" were told "no phone use." Obviously, I get the logic of this given that the phone was present for them but not for the "LS group." However, this could be a significant confound. Indeed, this could have drawn the participants attention to the fact that they couldn't use it and, in turn, could have distracted them, not simply because it was present but because of the fact that they were told they couldn't use their phone.

*** Thank you for this comment. The participants were informed to put their phones on silent, and either leave them at their side (HS) or hand them over to the researcher (LS). No phone use instruction was provided to both groups to prevent one group from accessing their phone over another. We have included this confound in the Discussion and suggested improvements. Please see revised section on page 20, line 369-373.

“Future studies could consider allowing participants to use their phone in both conditions and including eye tracking measures to determine their phone attachment behaviour.”

-Additionally, did the authors run any preliminary analysed based on how many participants were in each group when they participated? Given the importance just the mere presence of a cell is for the present study, the present of others could have influenced their results as well.
*** We thank the reviewer for this comment. We did not analyse for the presence of others as each session was mainly comprised of 3 participants only. We only had 2 sessions of 6 pax per session throughout.

Smaller Issues:

-How is the reader supposed to know what "phone conscious though" means in the abstract?

*** Thank you for highlighting this. Please see revised section on page 2, line 6.

-Pg. 2, Lines 13-14: A citation is needed to support this.

*** Thank you. We have added a new citation “GeekWire”. Please see the addition on page 3, line 14.

-Pg. 2 and throughout: "e.g." and "i.e." should only be used in parentheses. Otherwise, it should be "for example" and "that is" respectively and should always have commas around them.

*** Thank you. We have made the changes throughout.

-Pg. 2, Line 19: "Undoubtedly, the constant connectivity is applauded and desired..." This is way too editorial.

*** Thank you. We have revised the sentence (see below) and on page 3, line 18-19.

“Smartphones today have many functions that allows one to be constantly connected to others but this …”


-Pg. 3, Line 38: Describe what the "digit cancelation task" is
*** Thank you for highlighting this omission. We have added the following sentence on page 4, line 40-44.

“The digit cancellation task involves crossing out one digit from a series of numbers with reference to a target number. Performance is measured by referring to the number of lines completed and a cancellation score based on the total number of targets possible for the lines completed minus the number of errors made (failure to cancel a target or mistakenly cancelled an inappropriate number).”

-Pg. 3, Lines 41-42: "a mobile phone or a phone-sized notebook placed on participant's table before complete the tasks." Is not a complete sentence.
*** Thank you. We have revised the sentence to “…two groups; a mobile phone or a phone-sized notebook, which were placed on participant’s table before...” Please see page 4, line 46.

-Pg. 3, Line 42: "...showed no significant on..." Awkward. Reword
*** Thank you. We have revised the sentence to “…significance difference on performance between the phone and notebook condition for the simple digit ….” Please see page 4, line 47.

-Pg. 3, Line 43: Insert "the" between "during" and "simple"
*** Thank you. Please refer to the above comment as the sentence has been revised.

-Pg. 3, Line 52: "in" should be "on" (there are a lot of typos throughout. I won't highlight them all, but a careful proofreading is necessary
*** Thank you. We have engaged a native English speaker to proof read our revised manuscript.

-Pg. 3, Lines 54 & 57: Why do the authors provide the citation number to Ward et al., at the second instance and not the first?
*** Thank you for highlighting this. We have since revised this.

-Pg. 4, Lines 73-78: I think all those sentences could be integrated and stated much more succinctly

*** Thank you. We have made the changes. Please see page 5, line 77-83.

-Pg. 5, Line 89 and throughout: The authors use the term "memory" throughout. However, there are many different types of memory. They should specify what they mean exactly by "memory" at each instance.

*** Thank you. We have since included specific types of memory when describing past studies in earlier and subsequent pages.

-Pg. 5, Prior to "present study": I think the authors could do a better job of more explicitly stating what gap in the literature the their study will fill.

*** Thank you. We have included a research gap in our aim under Present Study. Please see section below, and also found on page 7, line 112-121.

“Prior studies have demonstrated the detrimental effects of one’s smartphone on cognitive function (e.g. working memory (13), visual spatial search (14), attention (12)), and decreased cognitive ability with increasing attachment to one’s phone (15,17,20). In addition to the presence of a mobile phone , it is also possible that one’s current affective state influences cognitive performance (21–23). But we are uncertain whether one’s current positive or negative affective / mood states plays a bigger role on cognitive function such as memory recall accuracy, suggesting a more complex relationship between current mood states and memory recall accuracy. To our knowledge, no study has examined the relationship between mood states and memory recall accuracy, with smartphone addiction and phone conscious thought as potential mediators. We hypothesised … “

-Results: Generally speaking, all t-tests should include cohen's d
*** Thank you. We have added Cohen’s d for all t-tests.

-Pg. 7, Line 138: "begun" should be "began."

*** Thank you. We have made the change.

-Pg. 8, Line 153 & 161: Technically, the 5 should be spelled out. However, at the very least, keep it consistent. That is, the authors us 5 and spell out six.

*** Thank you. We have made the change.

-Smartphone addiction Scale: Many of the sentences in this section have errors and need to be fixed. Additionally, the authors use "secondly" on line 167, but there's no "first" and there's no "third," etc... Also, examples of each of the sub-factors should be included.

*** Thank you. We have made the changes to include sample questions for each sub-factor. Please see page 10-11, line 190-216.

-Pg. 10: Some of this should be in the materials, not the procedure.

*** We have since relooked at our procedure and move out some items (e.g. phone conscious thought, and perception on learning) into Materials.

-Pg. 11, Gender: Why not include this analysis as a preliminary analysis. If gender, alternatively, is an important issue, then is should be set up as such in the lit review and the authors should examine the interaction with an F-test.

*** Thank you for this comment. Gender is not of interest in this study. However, we found that in our sample, females spent more time on their phone compared to males and wanted to determine if there is a gender effect on memory accuracy. We have included a preliminary analysis to include gender analysis under Results section. Please see page 14, line 255-258.

-Pg. 12, Lines 215-220: This should be a preliminary analysis. There's no reason to expect a difference between the groups assuming they were assigned randomly

*** Indeed, we agree with this comment that there should not be any difference. However, this analysis is more of a precautionary measure. Please see page 14, line 258-261.

-Pg. 13 (and elsewhere): The authors sometimes repeat the question in the results. This isn't needed. It's redundant.

*** We have now changed our sentences to better reflect our findings.

-Pg. 14: Why didn't the authors run an ANOVA to examine for an interaction between mood change and condition?

*** Thank you for this suggestion. We have not only added this, but also explained what is PA and NA difference. Please see page 17, line 298-303.

“We first computed the mean difference (After minus Before) for both positive ‘PA difference’ and negative affect ‘NA difference’. A repeated-measures 2 (Mood change: PA difference, NA difference) x 2 (Conditions: LS, HS) ANOVA was conducted to determine whether there is an interaction between mood change and condition. There was no interaction effect of mood change and condition, *F* (1, 117) = .38, *p* = .54, n_p_^2^ = .003. There was a significant effect of Mood change, *F* (1, 117) = 13.01, *p* < .001, n_p_^2^ = .10 (see Fig 2).”

-Pg. 15: More information is needed in terms of the variables included in the model.

*** Thank you. We have added more information about PA and NA difference score in the earlier results. Please see the above explanation.


-Pg. 18: There are no studies suggested under "Further Studies." The closest is a meaningless sentence: "Future studies should look into the online learning environment."

*** Thank you. What we meant is actually Implications, rather than Future Studies. We have now reworded the sub-heading.

-Pgs. 18-19: "These behaviors are likely to remain the same when students graduate and move into the workforce." Can the authors provide a citation to back this up or what are the authors basing this on?

*** We have revised this section. Please refer to page 21, line 387-395.

“The ubiquitous nature of the smartphone in our lives also meant that our young graduates are constantly connected to their phones and very likely to be on SNS even at work. Our findings showed that the most frequently used feature was the SNS sites e.g. Instagram, Facebook, and Twitter. Being frequently on SNS sites may be a challenge in the workforce because these young adults need to maintain barriers between professional and social lives. Young adults claim that SNS can be productive at work (33), but many advise to avoid crossing boundaries between professional and social lives (34,35). Perhaps a more useful approach is to recognise a good balance when using SNS to meet both social and professional demands for the young workforce.”

-Pg. 19, Lines 327-330: I don't understand this sentence or example...

*** We have reworded this section. Please see above.

-Pg. 19, Lines 342-343: "...the extent of the device purpose..." is awkward sounding.
Overall, many typos and awkward phrases. A careful proofreading is necessary.

*** We have revised it to “… integrate the phones into the classroom but will remain as a contentious issue between… “ See page 21, line 405.

Reviewer #2: 1. Is the manuscript technically sound, and do the data support the conclusions?

• How was sample size determined? Seems arbitrary, with no power analysis.

*** Thank you for this comment. We reported observed power of .66 in our findings, and effect size of ᶇ2 = .44. Please see the added content below and on page 8, line 134-136.

“Using G*Power v. 3.1.9.2 , we need 76 participants with an effect size of *d* = .65, α = .05 and power (1-β) = .8 based on Thornton et al.’s study, and 128 participants based on numbers from Ward’s study. “

• The addition of “phone-conscious thought” is a construct that does not seem to be validated in the peer reviewed literature. It’s ok to include this, but the methods behind the development of these questions should be clearly stated, and the authors must define this construct. There are some problems with how it is defined, because the question used relies specifically on phone-related thoughts during the task, while the phone is either in their presence (HS) or absent (LS). So, this question appears to serve as more of a manipulation check rather than a true measurement of phone-conscious thought. There are many issues with the construct of “phone-conscious thought” in the current manuscript.

*** we thank the reviewer for this insightful comment. We certainly did not intend this question to be a manipulation check about their phones. We acknowledged that we omitted a fair amount of phone conscious thought in our earlier submission. We have since added more information about phone conscious thought in Abstract (page 2, line 6), Introduction (page 6, line 91-97), and Discussion (page 19, line 347-353).

• Why is affect measured both before and after the memory test? Explain the rationale. Is the memory test expected to influence mood in any way?

*** we thank the reviewer for this comment. We realised that this is a major oversight on our part. The main reason for including affect measurement before and after was derived on the possibility that one’s mood may affect your cognitive function, and not simply due to phone presence. We have since made this clearer under Present Study (refer to page 7, line115-121 and in Results (refer to page 16-17, line 295-309).

• The inclusion of the phone-conscious thought question in the beginning of the study may have primed participants to think about their phones more overall, and this may have inflated the differences between the LS and HS groups.

*** we thank the reviewer for this comment. The phone conscious thought was asked at the end of the memory task. This was included in the Procedure section, page 12, line 243.

• There should have been a 3rd control group where participants were given no instruction about what to do with their phone. This would help assess whether the LS group experienced lower recall or if the HS group experienced higher recall, relative to baseline.

*** We thank the reviewer for this comment. One of the objectives in this study to examine the effect of a phone presence when participants are completing a simple learning and memory task. For this objective, we had two conditions; phone present (HS) and phone absent (LS). By having a third control with no instructions on what to do with the phone is addressing a different objective and that’s not part of our study objectives. We acknowledged that instruction on phone use may possibly be a confound and as such, we have addressed this limitation in our Discussion (see page 20, line 371-373).


*2. Has the statistical analysis been performed appropriately and rigorously?

• Results for the affect/mood changes are very unclear and should be edited to be more precise. Needs to be much more descriptive.
*** We noted this. We have since revised this section. Please see page 16-17, line 295-309

*3. Have the authors made all data underlying the findings in their manuscript fully available?

Yes


*4. Is the manuscript presented in an intelligible fashion and written in standard English?

The writing is unclear at times with strange vocabulary choices (e.g. “Undoubtedly, the constant connectivity is applauded and desired but this has also spiralled into an obsession with the device for many individuals” lines 19-20). What do the authors mean by “applauded and desired”? Further, writing around the explanations of the relevant literature is imprecise and should be cleaned up so that no previous findings can be mischaracterized. Requires rigorous editing to be publishable, in my opinion.

*** We thank the reviewer for this comment. We have engaged a native English speaker to proofread our manuscript in accordance with academic writing practices.

Lines 57-58: In which direction? And in which tasks? All of them? Needs much greater precision.

*** we thank the reviewer for this comment. We have revised this sentence to better inform the reader on Ward et al.’s findings.
