# Supplementary material for: Mobile phones: The effect of its presence on learning and memory
Source: PLoS One. 2020 Aug 13;15(8):e0219233. doi: 10.1371/journal.pone.0219233 (PMC7425970; doi:10.1371/journal.pone.0219233)
Supplement: Attachment — Submitted filename: Response to Reviewers PONE-D-19-17118R1 Yong.docx [file pone.0219233.s002.docx]

Friday, 24 April, 2020

PONE-D-19-17118R1
Mobile phones: The effect of its presence on learning and memory
PLOS ONE

Dear Editor,

We truly appreciate the comments made by the reviewers and have since made the changes as recommended. We have secured another reviewer and proof-reader to reorganise and rearrange our sentences to make the manuscript more coherent. Please see line-by-line feedback on each comment marked with ***. Please note that the line numbers and page numbers mentioned below are referenced to the clean version ‘Manuscript’.

Thank you.

Best wishes,

Min Yong & Clarissa Tanil

----

Reviewer #1: Overall, I congratulate the authors on the revisions they've made already. The paper is much better for it.

However, I still have some concerns.

Most notably, as far as I can tell, the contribution this manuscript makes to the literature is in the inclusion of emotion. Indeed, the authors make this point quite clear in their "Present Study" section. Indeed, they state "But it is uncertain whether one's current positive or negative affective/mood states plays a bigger role on cognitive function..." but this doesn't seem to cohere very well with the rest of the paper. Barely anything is mentioned about emotion in the intro/lit. review (till the very end). In the analyses, the stats including emotion seem almost like an afterthought. Additionally, emotion is barely mentioned in the discussion. I realize that the authors found no statistical difference across groups, and therefore don't focus on them, but that raises another possible issue: interpreting a null result. If the primary motivation for this study was emotion, it seems to me that one would devise a different design whereby you also manipulate emotion and then examine the different conditions in terms of mobile phone salience.

Thus, at the heart of it, the present paper replicates prior research and then finds a null effect for their primary research question, making interpretations difficult.

*** We thank you for this. We do agree that we have omitted a fairly huge amount on the affective state and have since revised the Introduction to explain the interactions between emotion and cognition. Please see Page 4-5, Line 60-73 in Introduction. We have also reorganised the Aim/Hypotheses section – please see Page 5-6, Line 75-92.

For these reasons, I, unfortunately, am recommending rejection.

For the authors reference moving forward, the paper was still a bit hard to parse in places due to language issues throughout. I know the authors state that a native English speaker proofread it, but more diligent proofreading is needed in the future.

*** We do apologise for this and have since secured a second proof reader. We hope that the manuscript is far more legible now. We have also reorganised parts of the manuscript to make it more coherent.

----

Reviewer #3: The experiment presented in this paper is aimed at primarily investigating whether the salience of a phone (high vs. low) impacts memory accuracy. It is a fairly straightforward experimental design and set of results. My main concern is that the paper lacks a clear mechanism to explain the results. Is the main result (i.e., HS leads to lower memory accuracy than LS) due to the fact that high salience participants are distracted during encoding? Is it due to retrieval deficits? Do they not consolidate the information properly? Is it evidence of a bandwidth effect, by which phone-related thought intrusion interferes with memory processes?

My sense is that not only that the experimental design did not attempt to answer the question mechanistically, but there is no attempt to theoretically scaffold the results in a potential mechanism. I would advise the authors to at least speculate as to what could explains the set of results they obtained and to hint at possible investigation of the mechanism involved.

*** Thank you for this feedback. We will first address the question on whether the memory recall accuracy was due to encoding, consolidation or retrieval failure. From previous studies, simple versions of a cognitive task was not an issue between low salience (LS) and high salience (HS) participants (1–3) for they had similar performance levels. This suggests it is unlikely that participants had problems at either encoding, consolidation or retrieval for the simple tasks.

However in our study, we used OS Span task which is considered a complex task compared to simple memory span (4). Although we did not include simple memory span as a contrast to OS Span, previous studies suggest that this is not necessary because of similar performance levels across conditions. One of our aims was to replicate a previous study in investigating whether the presence of a smartphone was sufficient to affect memory recall accuracy (5). We found that our participants had significant difference in memory recall accuracy between HS and LS conditions, *p* = .02. While our results concurred with previous study findings, we are unable to tease apart whether the presence of the smartphone had interfered with encoding, consolidation, or recall phase in our participants. However there is a possibility that the separation from their smartphones may have caused feelings of anxiety, and anxiety may interrupt memory consolidation as suggested by some (6,7). This is certainly something of consideration for future studies.

Second, to the bandwidth effect interfering memory processes, we suspect that this might be the case, rather than an issue of failure in a specific memory process. This is because participants with smartphones or texters could generally perform simple cognitive tasks as well as those without, and the presence of the smartphone next to the participant is responsible for the increase in cognitive load (1,3,5).

Other than the smartphone presence to increase cognitive load, we intended to manipulate participants’ affective state by prohibiting smartphone usage (HS) or taking it away (LS). Previous research has shown that experiencing positive affect (PA) or negative affect (NA) would influence cognitive performance (6–8). We predicted that the short-term separation from smartphone would evoke some anxiety, measured either having lower positive affect (PA) or higher negative affect (NA) post-test. We also predicted that separation from the phone is directly correlated to lower memory recall (LS condition) (part of hypothesis 2). An increase in NA or decrease in PA (as an indicator of separation anxiety to their smartphones) often have a negative effect on cognition (6,7). Further, one study shown an increased level of anxiety even in 10 minutes (9) and OS Span generally takes 20 minutes. Our results supported this hypothesis for LS participants who experienced a stronger negative affect had poorer memory recall accuracy (*r_s_* = -.394, *p* = .002, *n* = 58). This suggests that phone separation anxiety does increases cognitive load. We did not find any significant relationship between NA and memory recall accuracy for the HS participants and also for the PA difference in both groups (see Results, page 14-15, line 259-265).

We also examined another variable – phone conscious thought – described in past studies (3,5). Here, we found that phone conscious thought is negatively correlated to memory recall in both HS and LS conditions (see Results page 15, line 273), and uniquely contributed 19.9% in our regression model.

Taken together, the results showed that phone conscious thought is a significant contributor to the bandwidth effect interrupting their memory processes, and not the change in affective states as we had originally predicted. We do not think that participants’ memory failed at critical points e.g. encoding, retrieval, consolidation. Our participants memory processes are not likely to be impaired as they are neurotypical young adults, unlike well-documented cases in ageing or traumatic brain injury populations. In conclusion, the presence of the smartphone and frequent thoughts of their smartphone were contributors that interrupted their memory processes.

We do acknowledge several limitations in our study. First, we did not ask the phone conscious thought at specific time points in this study. Having done so might determine whether such thoughts hindered encoding, consolidating, or retrieval. Second, we did not include the simple version of this task as a comparison to rule out possible confounds within the sample. We did maintain similar external stimuli in their environment during testing, e.g. all participants were in one specific condition, lab temperature, lab noise, and thereby ruling out possible external factors that may have interfered with their memory processes. Third, the OS task itself. This task is complex and unfamiliar, thus may have caused some disadvantages to some. However, the advantage of this task being likely to be more unfamiliar – requiring more cognitive effort to learn and progress – demonstrates the limited cognitive capacity in our brain, and whether such limitation is easily affected by a smartphone presence.

References

1.	Ito M, Kawahara J-I. Effect of the presence of a mobile phone during a spatial visual search. Jpn Psychol Res. 2017 Apr 1;59(2):188–98.

2.	Bowman LL, Levine LE, Waite BM, Gendron M. Can students really multitask? An experimental study of instant messaging while reading. Comput Educ. 2010 May 1;54(4):927–31.

3.	Thornton B, Faires A, Robbins M, Rollins E. The mere presence of a cell phone may be distracting: Implications for attention and task performance. Soc Psychol. 2014;45(6):479–88.

4.	Francis G, Neath I, VanHorn D. CogLab On A CD, Version 2.0. Belmont, CA: Wadsworth; 2008.

5.	Ward AF, Duke K, Gneezy A, Bos MW. Brain drain: The mere presence of one’s own smartphone reduces available cognitive capacity. J Assoc Consum Res. 2017 Apr 1;2(2):140–54.

6.	Levine LJ, Lench HC, Karnaze MM, Carlson SJ. Bias in predicted and remembered emotion. Curr Opin Behav Sci. 2018 Feb 1;19:73–7.

7.	Okon-Singer H. The role of attention bias to threat in anxiety: mechanisms, modulators and open questions. Curr Opin Behav Sci. 2018 Feb 1;19:26–30.

8.	Gray JR. Integration of Emotion and Cognitive Control. Curr Dir Psychol Sci. 2004 Apr 1;13(2):46–8.

9.	Cheever NA, Rosen LD, Carrier LM, Chavez A. Out of sight is not out of mind: The impact of restricting wireless mobile device use on anxiety levels among low, moderate and high users. Comput Hum Behav. 2014 Aug 1;37:290–7.
