# Supplementary material for: Mobile phones: The effect of its presence on learning and memory
Source: PLoS One. 2020 Aug 13;15(8):e0219233. doi: 10.1371/journal.pone.0219233 (PMC7425970; doi:10.1371/journal.pone.0219233)
Supplement: Attachment — Submitted filename: Response to Reviewers PONE-D-19-17118R2 Yong.docx [file pone.0219233.s003.docx]

Monday, 13 July, 2020

PONE-D-19-17118R2

Mobile phones: The effect of its presence on learning and memory

PLOS ONE

Dear Editor,

Thank you for the comments and suggestions. We have changed the manuscript as recommended. Please see line-by-line feedback on each comment marked with ***. Please note that the line numbers and page numbers mentioned below are referenced to the clean version ‘Manuscript’.

Thank you.

Best wishes,

Min Yong & Clarissa Tanil

---

Editor:

Dear Editor, we found that the line numbers references differ from clean_manuscript_v3 and the one listed in your comments. We note that your line number reference is +1 from what was included in the manuscript, and will be using this formula to address your concerns. We do apologise if we did get the line numbers wrong.

1. The main issues concern the introduction. One phrase in Line 62 has no verb and therefore can not be a sentence. You need to correct the grammatical structure.

*** We thank the editor for pointing this out. We have since removed the sentence.

2. Secondly it would be good if the introductory sentence to the paragraph beginning on line 61 indicated the relationship between cognition and affect is important for understanding the impact of mobile phone use on memory. As it is written the paragraph does link well to the previous paragraphs.

*** We thank the editor for this comment. We have revised the paragraph, please see Line 60-61, page 4.

“Further, we need to consider the relationship between cognition and emotion to understand how frequent mobile phone use affects memory e.g. memory consolidation. Some empirical findings … “

3. Further line 82 makes reference to smart phone addiction very briefly but the discussion focuses a great deal on smart phone addiction. You need to define smart phone addiction and indicate why it is important to examine this construct in your study.

*** We thank the reviewer for omission on our part. Please find the newly added sentences below on Line 83-88, page 5-6.

“One in every four young adults is reported to have problematic smartphone use and this is accompanied by poor mental health e.g. higher anxiety, stress, depression (Sohn et al., 2019). One report showed that young adults reached for their phones 86 times in a day on average compared to 47 times in other age groups (Deloitte Development LLC, 2017). Young adults also reported that they “definitely” or “probably” used their phone too much, suggesting that they recognised their problematic smartphone use. “

4. Further you need to define the subscales in of the SAS in the methods and also justify under phone consious thought why you are including this question.

*** We thank the reviewer for this comment. Please see the inclusion for SAS subscales on Line 159-166, page 9.

“SAS contained six sub-factors; daily-life disturbance that measures the extent to which mobile phone use impairs one’s activities during everyday tasks (5 statements), positive anticipation to describe the excitement of using phone and de-stressing with the use of mobile phone (8 statements), withdrawal refers to the feeling of anxiety when separated from one’s mobile phone (6 statements), cyberspace-oriented relationship refers to one’s opinion on online friendship (7 statements), overuse measures the excessive use of mobile phone to the extent that they have become inseparable from their device (4 statements), and tolerance points to the cognitive effort to control the usage of one’s smartphone (3 statements).”

We have also added the justification to the phone conscious thought. Please see this inclusion on Line 173-175, page 9.

“The aim of this question was two-fold; first was to capture endogenous interruption experienced by the separation, and second to complement the smartphone addiction to reflect current immediate experience.”

Reviewer #3: The authors addressed my comments satisfactorily, so I recommend acceptance for the manuscript. I doubt, though, that anxiety plays a huge role in these dynamics, given that it is hard to imagine that one would be able to create the levels of anxiety necessary for the disruption of cognitive function by simply temporarily removing their phones.

***

5. How frequent do we reach for our phones?

*** Surprisingly, high, but unsurprisingly higher for young adults. Deloitte 2017 survey reported that the average American reaches for their phones 47 times while young adults (aged between 18 to 24) reach for 86 times. The same survey also reported that 89% looked at their phone within an hour of waking up and that 81% also looked at their phone within an hour before going to bed. Further, 90% of young adults reported using their phones in their daily activities ranging from shopping, leisure time, talking to friends, crossing the road, and this trend has been consistent for the past three years. The young adults also reported that they “definitely” or “probably” use their phone too much, suggesting some form of recognising their addiction (Deloitte Development LLC, 2017). Another poll reported that one in every 10 Americans check their phones every four minutes, and that most people struggle to go beyond 10 minutes without checking their phone (SWNS, 2017).

We have added couple of sentences to further highlight on mobile phone addiction under Research Aim (see Line 85-88, page 5-6).

**References**

Deloitte Development LLC. (2017). *2017 Global mobile consumer survey: US edition. The dawn of the next era in mobile* (p. 18). https://www2.deloitte.com/content/dam/Deloitte/us/Documents/technology-media-telecommunications/us-tmt-2017-global-mobile-consumer-survey-executive-summary.pdf

Sohn, S., Rees, P., Wildridge, B., Kalk, N. J., & Carter, B. (2019). Prevalence of problematic smartphone usage and associated mental health outcomes amongst children and young people: A systematic review, meta-analysis and GRADE of the evidence. *BMC Psychiatry*, *19*(1), 356. https://doi.org/10.1186/s12888-019-2350-x

SWNS. (2017, November 8). Americans check their phones 80 times a day: Study. *New York Post*. https://nypost.com/2017/11/08/americans-check-their-phones-80-times-a-day-study/
